# Operational mental health. . .what practitioners and commanders should know

**DOI:** 10.1177/00207640241261208

**Published:** 2024-07-24

**Authors:** Martin Deahl

**Affiliations:** King’s College London Institute of Psychiatry Psychology & Neuroscience, London, UK

**Keywords:** Military mental health, occupational psychiatry, military medicine, Field mental health team

## Abstract

**Background:**

Mental Health support to military operations is well established as an integral part of military medicine. Unfortunately, Commanders often receive little or no training in how best to use their mental health assets or what their capabilities are. Conversely, members of a Field Mental Health Team frequently have no operational experience and try to merely translate their civilian practice onto the battlefield.

**Aim:**

This article describes what mental health professional can, and should do on military deployments and calls for greater training and awareness of both Mental Health professionals and Operational Commanders to foster mutual understanding and use the Field Mental Health Team to best effect.

**Method:**

The paper drawson the experience of working in a Field Mental Health Team on six operational deployments in Iraq and Afghanistan.

**Results:**

Military mental health professionals work mostly in peacetime and this work ill prepares them for the very different type of work required of them on operations.

**Conclusion:**

More training is required to prepare both practitioners and commanders for the mental health issues that confront them on operational deployments.

Mental Health support to Operation deployments is too often a little discussed and easily overlooked issue yet mental health professionals can play an important role not only in the Field, but also in the pre and post deployment phases of Operations. Doctors, psychologists, nurses and medics who may be having their first experience of an Operational deployment are often ill-prepared for their role, as indeed are Commanders who are frequently unaware of how best to deploy and utilise their mental health assets to best effect. Too often the geographic location of the mental health department within a Field Hospital symbolises attitudes to mental health in general. . ..at the rear of the hospital complex, out of sight and out of mind. All this belies the potentially invaluable role that mental health professionals can play, which can go well beyond treating individual referrals, with a much broader role, improving Operational efficiency and effectiveness in Theatre, as well as making a potentially important contribution to the pre and post deployment phases of an operation, boosting both Unit morale as well as the mental health of serving-personnel and Veterans, and indirectly enhancing the image and reputation of the Unit in the wider public consciousness.

## The commanders’ dilemma

What to do with psychiatric casualties on the battlefield poses a perennial dilemma for commanders who traditionally have received little or sometimes no training in this area. . .In my own experience, on a UK Commanding Officer’s Designate course, mandatory for all prospective military Commanders, 3 days of the 14-day course was devoted to different ways of disciplining malfeasant soldiers, but nothing in relation to the psychological welfare of troops or dealing with mental breakdown on the battlefield. As a result, commanders bring their prejudices with them on Operations and, regardless of the advice they may receive from mental health professionals, decisions regarding the criteria for becoming a psychiatric casualty on the battlefield ultimately lies with the Commanding Officer. Medical Officers may advise, but the Commanding Officers temperament, personal prejudices and the tactical situation often carry more weight than the views of a medical officer or mental health professional. The Commanding Officers perennial dilemma is that if the threshold is set too low, there is the possibility of a mass exodus from the battlefield, after all, who in their right mind would risk their lives and fight on, if they knew there was an easy, and honourable way out? Unit morale mitigates this, but only to a point. On the other hand, setting the threshold too high risks psychologically impaired soldiers in combat who are not only militarily ineffective, but also potentially undermine morale and unit cohesion, creating more problems than their contingent presence justifies.

The two World Wars teach an interesting lesson; in the First World War all protagonists set strict and often harsh criteria to define a psychiatric casualty and many soldiers on all sides were executed for cowardice or desertion. The incidence of psychiatric breakdown was roughly the same on all sides, and in the UK for example, more than 3,000 death sentences were passed and 346 UK servicemen were executed, many of these undoubtedly mentally ill. By the time of the Second World War attitudes in the UK and US had softened, whitest German and Soviet forces maintained the same harsh criteria used in the First World War. The incidence of psychiatric breakdown remained at 9 per 1,000 in German and Soviet forces, whitest unsurprisingly it rose to 39 per 1,000 amongst US and UK forces. What was interesting was that there was a proportionate increase in rates of disease and non-battle injuries (DNBI), potentially self-inflicted by neglect or directly self-inflicted such as Trench Foot, Frostbite, accidental gunshot wounds in the German and Soviet forces, that offset the reduced number of psychiatric casualties, that is to say, exactly the same numbers of soldiers on all sides were being evacuated, but German and Soviet troops resorting to other means avoiding the potential for harsh punishment by their Chain of Command and the opprobrium of their peers. It appears that soldiers who were unable to function or cope in combat, and who were denied the possibility of becoming a psychiatric casualty, ‘broke down’ in other less obvious and more institutionally acceptable ways. The overall numbers of casualties remained the same; only the manner of their presentation varied (Gabriel, 1987).

Military psychiatry is notorious for its collective short-term memory and having to re-discover the lessons, hard learned, from previous conflicts. Good practice on the Battlefield is learned incrementally and only comes with operational experience. Today’s military mental health professionals all too often, deploy on operations expecting to simply translate their civilian practice into the Operational arena and the specific skills of military psychiatry in the Field have to be learned, often painfully, by trial and error on the job.

## On the battlefield

The mental health and wellbeing of service personnel is immeasurably improved if they receive effective, timely care whilst still in service, at the point of injury. It is worthwhile, therefore, to reflect on aspects of care delivery not commonly taught or found in textbooks or instruction manuals.

Clearly prompt access to medical officers or psychologists and nurses, properly trained to recognise combat stress reactions is key, however, chaplains and other welfare personnel have a role to play and should also receive training in the recognition of mental disorders as well as being prepared to work closely with the Field Mental Health Team. Similarly, General Duties medical officers should have ready access to mental health professionals to enable timely referral and advice. Given a relative lack of training and reliance on personal proclivity and whim, it behoves medical officers and mental health professionals to establish good working relationships with commanders to foster mutual respect and understanding, pre-empting potential conflict and disagreement over individual cases. This working relationship should begin in the pre-deployment training phase of Operations and should not wait until the protagonists have been deployed in the Field.

Ideally, the Unit senior mental health professional should attend operational briefings both to increase mutual contact and familiarity with Unit Commanders, but also, especially, to give themselves a better appreciation of the bigger picture to enable them to put their work in context and better understand the conflicting pressures facing the Chain of Command. The workaday routine of mental health professionals goes well beyond seeing individual referrals; for example, current western initiatives such as TRIM (Trauma Incident Risk Management) remain problematic and raise many unanswered questions requiring specialised mental health advice and support; TRIM practitioners focus on a specific, single event trauma, but what to do when individuals have faced multiple, daily traumatic events? It is also often necessary to remind the Chain of Command that many soldiers suffering from apparently serious psychological symptoms (including psychosis) are also physically exhausted, extremely tired and dehydrated, or in physically poor condition and if operationally possible, simple measures such as allowing these individuals to receive respite, adequate rest, sedation when necessary, comfort and nutrition, itself can be curative. The Operational Welfare Package which enables soldiers access to internet and free mobile phone time, is itself problematic and can become a double-edged sword; domestic problems back home which, in days gone by could take weeks to be communicated to a soldier in Theatre by ‘bluey’ or post, by which time the situation had usually resolved itself, are today known instantaneously and in real time thanks to modern technology. Life on operations was once said to be simple, unfettered by the complexities of modern living, you simply turned up on time and didn’t even have to worry about what to wear. Today, soldiers are confronted with their domestic crises, by phone or on-line, in the Field in real-time, by an angry spouse or partner, who sees her partners absence as contributory to the crisis, appropriating blame accordingly. It gets worse; during my Command in Iraq I had to intervene on six occasions with banks, building societies and loan companies threatening soldiers with repossession of their home, or legal action for non-payment of loans or debts (often because of a hiatus in salary payments due to the deployment itself – the Army’s fault). Admittedly, the agencies involved became sympathetic and much more conciliatory to their clients when they discovered their situation and where they were emailing from, but had I not learned from past experience that the Operational welfare Package was not without its own problems, I would have been none the wiser and my troops would not of received the support necessary to quickly resolve their dilemma.

On top of all this are the inevitable individuals who passed through pre-deployment medical checks failing to declare pre-existing illnesses and running out of medication, typically medication not on manifest, or readily available in Theatre. Then, of course are the workaday stresses and tensions that inevitably arise in any close-knit community which have nothing to do with traumatic events but can nevertheless escalate into mental health problems. No matter what prohibitions are put in place by the Chain of Command sex, drugs, alcohol and relationship difficulties are part of the human condition and almost inevitably follow troops into Theatre with the potential to cause considerable damage both the individual as well as the Unit.

## Homeward bound and falling off the cliff edge

Efforts to identify psychological problems on return from operations, although laudable, are seldom effective. The author has first-hand experience of groups of homecoming soldiers in one large room, being asked by a rather intimidating Warrant Officer. . .‘*Anybody with psychological issues*?’ The responses were predictable!

Returning to ‘normal’ life after Operations can be both challenging and traumatic. The physical, psychological and emotional gulf between life on Operations and the civilian world is enormous, unfathomable to those who haven’t personally undergone the experience. The experience of troops returning from Operations is akin to falling off a cliff edge. . ..anything that smooths and enables a more gradual transition (transforming the cliff from a vertical precipice into a more gradual ‘slope’ is to be welcomed). Acutely, personnel returning from operation are often exhausted, dirty and emotionally still in an adrenaline fuelled war-fighting mode. Operational decompression, allowing personnel to rest and recuperate for a few days before returning home allows them an opportunity to process a shared experience with comrades, regain composure and physically refresh before returning to loved ones and the ‘real world’ (Hacker-Hughes et al., 2008).

As well as the obvious physical needs of returning combatants it is important to bear in mind the extraordinary responsibilities that can go with the most junior ranks. Status and identity are lost on return from operational deployment which can itself create, or exacerbate mental health problems. This is all the more so for Reservists and Regulars leaving service and re-entering the civilian world. . .as one General opined. . .‘*you go from being a somebody to a nobody, overnight’*.

Education on demobilisation, particularly in relation to excessive alcohol, drug use and ‘risky’ behaviour may be useful, but only if delivered by someone with perceived credibility in the eyes of the audience. Information about benefit entitlements, and support and services available post discharge are equally important taking care to deliver this information in an easily understood format, too often servicemen are anxious to get home pay little attention to verbal information at the point of discharge. Delivering information at the right time and place can be problematic. On one occasion when the author’s efforts to provide literature on post operational mental health reactions and support by placing a specially prepared one side of A4 handout on the seat of returning transport aircraft home, and with the full authority of the (GoC) General Commanding Operations in Theatre, the initiative was blocked by higher authority on the grounds that it would require a change to the Air Navigation Order.

The Unit, the Regiment and the Corps are perceived by many servicemen as a family and wherever possible there should be mechanisms for maintaining links for those who wish with both the wider military as well as former comrades: Regimental associations, veteran’s networks promoting social contact can be more effective than specific therapies for veterans. Finally, the manner in which a serviceman leaves the military can be crucial to mental wellbeing and an appropriate fulsome ‘*goodbye, well done and thank you*’. from, once again, a senior military figure with perceived credibility, ideally the Commanding Officer, can make all the difference in the adjustment to civilian life and the ability to tolerate and cope with psychological symptoms.

The conclusion of an Operational Tour and handover is often chaotic and characterised by everybody’s efforts, from the Commanding Officer downwards, to get home as quickly as possible. Opportunities for existing incumbents to handover to their successors may be limited or non-existent due to constraints beyond their control, such as the availability of transport and a multitude of other competing commitments. In my own multiple operational deployments, both as Psychiatrist and Unit Commanding Officer, not once did I experience a satisfactory handover from my predecessor.

The manner in which service-personnel part company with their peers and commanders is crucial to their subsequent well-being and long term attitudes towards the military. Soldiers need to return from Operations with a ‘feel-good’ factor and feel valued and appreciated for their efforts. Efforts to ensure that they receive a fulsome ‘goodbye and thank you’ from the Commanding Officer can make the difference between becoming a psychiatric casualty or not, as well as shaping lifelong attitudes to the military for the next generation of service veterans, arguably the most important ambassadors for the Armed Forces in the civilian world. The return from Operations is often un-orchestrated and chaotic but raising the awareness of Commanders so they factor in both the time and opportunity to thank their personnel making them feel that their contribution is valued, should be a given, and not a luxury. This can be particularly important for Individual Reinforcements and attached personnel, many of whom will have established close working relationships with their comrades and who may never see them again.

Post-operational decompression can play an important role in this process. In days gone by, troops returned from operations mostly on ships and had days or weeks on board to adjust from an adrenaline fuelled lifestyle to the relative mundanity of everyday life. This prescribed ‘time-out’ not only gives soldiers a chance to say goodbye to comrades, but allows rest, an opportunity for adequate personnel administration, as well as the chance to process often traumatic experiences, informally, with comrades. . ..they effectively decompress themselves. Now, without a built in period of ‘decompression’ even the farthest flung operational theatres are just a few hours flying time from home. There is a considerable literature and evidence base as to the benefits of 3 or 4 days of decompression, however, even the best laid plans can go awry if the process is not carefully managed; in particular, the timings for events during the decompression process should be flexible and take account of shifting travel times. As Commanding Officer it was bizarre as it was unedifying to discover the troops time for swimming and beach-relaxation taking place in darkness at 0300 simply because of altered flight arrangements and a delayed arrival. Even worse was an enforced cinema visit that followed at 0430, nonsensical yes, but the programme had to be followed at all costs.

Operational Decompression should not be the end of the post-tour engagement with comrades. A post tour Reunion 3 to 6 months following a Unit’s return is all too often *ad hoc* and driven by the enthusiasm of individual Commanders. These should be an integral part of the Operational Unit SOP and be treated as a given, and not a luxury. Importantly, they should not be confined to the parent Unit and should be open to all, everybody who deployed should be invited, including Reservists I.R.’s and Individual Reinforcements. This not only depends on the operational Commander, but also the collaboration and cooperation of the parent units who ‘own’ I.R’s and Reservists.

In the longer-term, Regimental Associations have a role to play supporting Veterans and helping promote contact with the ‘Regiment’ as well as fostering community links. At present there is no standard operating procedure (SOP) for a Regimental Association and their quality varies dramatically from that of little more than a senior officers dining club to, at the other end of the spectrum, a care and social welfare service with drop-in facilities and pro-active outreach to members. These arrangements should be standardised with clear guidelines and an explicit statement of what the expectations of the Regimental Association should be. Retaining links with the military not only provides a vehicle for social contact and a welfare safety-net for ex service personnel struggling in the civilian world, but can be exploited as a platform for the detection of delayed-onset of post traumatic disorders. Combat related mental health problems may fail to present for many years (e.g. In the UK there is a 14.1-year delay between discharge from military and presentation to specialist veteran’s mental health services) (Fletcher, 2007) and it is well established that 10% of PTSD cases are delayed in onset and presentation (Utzon-Frank et al., 2014). Mental illness and suicide more likely in young (<25), single male servicemen, who also happen to be the individuals least likely to seek help (Crawford et al., 2009). Stigma and reluctance of men to admit mental health problems remains a common barrier to treatment. The displacement of suffering and anger into alcohol, drugs and domestic and sexual violence is commonplace on return from operations, as is, so called ‘Risky’ behaviour such as reckless driving, gambling, fighting, sexual promiscuity, all of which are of course fuelled by excessive alcohol misuse (Killgore et al., 2008).

The Regimental Association has a potentially important role to play enabling contact between serving former comrades and is a potentially important means of enabling the early detection of combat-related problems, not least because a shared background facilitates discussion and openness about combat experience that often remains untold and hidden in a civilian context.

In conclusion, military mental health professionals have much to offer, not just as therapists, but as educators, facilitators and networkers, both within the Unit, between the Unit and Chain of Command, the veteran and civilian mental health services, and between service personnel themselves, helping to create a platform to promote the shared experiences and enduring friendships that emerge from the shared experience of an Operational deployment

The Regimental Association has a potentially important role to play enabling contact between serving former comrades and is a potentially important means of enabling the early detection of combat-related problems, not least because a shared background facilitates discussion and openness about combat experience that often remains untold and hidden in a civilian context.

In conclusion, military mental health professionals have much to offer, not just as therapists, but as educators, facilitators and networkers, both within the Unit, between the Unit and Chain of Command, the veteran and civilian mental health services, and between service personnel themselves, helping to create a platform to promote the shared experiences and enduring friendships that emerge from the shared experience of an Operational deployment.
